# Perceived Changes in Health-Seeking Behaviour Following the COVID-19 Pandemic: A Qualitative Study of Urban and Rural Populations in Eastern Gujarat

**DOI:** 10.7759/cureus.117837

**Published:** 2026-10-13

**Authors:** Salil Sharma, Niraj Pandit, Nirmal Jyoti Jyotsana, Srishti Mishra

**Affiliations:** 1 Community Medicine, Smt. B.K. Shah Medical Institute and Research Centre, Sumandeep Vidyapeeth Deemed to be University, Vadodara, IND

**Keywords:** covid-19, eastern gujarat, financial barriers, healthcare decision-making, health seeking behaviour, post-pandemic behaviour, qualitative research, urban–rural

## Abstract

Background and objectives

The coronavirus disease 2019 (COVID-19) pandemic significantly disrupted healthcare systems and altered patterns of healthcare utilization worldwide. In India, where structural inequities in healthcare access persist, the pandemic may have influenced health-seeking behaviour differently across urban and rural populations. Understanding these behavioural shifts is critical for strengthening health system preparedness in future public health emergencies. This study aimed to explore, using a qualitative descriptive design, participants’ perceived changes in health-seeking behaviour, healthcare preferences, and decision-making following the COVID-19 pandemic among adults aged 18-85 years living in urban and rural areas of eastern Gujarat, India.

Methods

This descriptive qualitative study was conducted as part of a larger mixed-methods investigation in eastern Gujarat between October 2023 and April 2025. Face-to-face, semi-structured, in-depth interviews were conducted with 20 purposively selected participants (10 from urban Vadodara and 10 from rural Sankheda taluka, Chhota Udaipur district). Interviews were conducted in Gujarati or Hindi, audio-recorded, and translated into English. The interview guide was pilot-tested and refined before data collection and was informed by the PASS model and relevant literature. Data were analysed using inductive thematic analysis, with codes organized into categories and themes through iterative review.

Results

Five major themes emerged. Participants described perceived changes in health-seeking behaviour following the COVID-19 pandemic. These included greater preference and trust in formal healthcare, alongside a continued preference for private facilities despite financial constraints. Healthcare decisions remained influenced by affordability, accessibility, perceived quality of care, symptom severity, and family considerations. Self-medication, home remedies, and other traditional systems of medicine continued to be used, particularly for minor illnesses. Telemedicine was viewed positively by some participants but had limited uptake. Participants also described greater attention to hygiene, diet, exercise, and other preventive practices.

Conclusions

Participants described perceived changes in health-seeking behaviour following the COVID-19 pandemic, including greater trust in formal healthcare and increased attention to preventive practices. However, financial constraints, accessibility barriers, perceived delays in public facilities, and continued self-treatment for mild symptoms remained important influences on healthcare decisions. These findings provide contextual insights into healthcare decision-making in the selected study settings.

## Introduction

The coronavirus disease 2019 (COVID-19) pandemic, caused by the severe acute respiratory syndrome coronavirus 2 (SARS-CoV-2), represented an unprecedented global public health crisis that profoundly affected healthcare systems and population behaviour worldwide. Beyond its direct morbidity and mortality, the pandemic disrupted routine healthcare delivery through lockdowns, mobility restrictions, and the reallocation of health resources, thereby altering patterns of healthcare access and utilization. These disruptions were particularly significant in low- and middle-income countries such as India, where health systems already face constraints related to accessibility, affordability, and workforce capacity [1,2].

Health-seeking behaviour refers to actions undertaken by individuals when they perceive a health problem or seek to maintain well-being. It is a multidimensional process shaped by sociocultural norms, economic conditions, perceived illness severity, healthcare availability, and previous experiences with the health system [3,4]. Evidence suggests that pandemics can act as critical turning points, influencing healthcare utilization through both positive changes, such as increased hygiene practices and health awareness, and negative responses, including delayed care-seeking and increased reliance on self-medication [5].

In India, Substantial qualitative and quantitative evidence has been documented in the subcontinent suggesting disruption of healthcare access, economic consequences, and limited but emerging use of teleconsultation during the COVID-19 pandemic, highlighting existing inequities in urban and rural populations. While urban areas experienced higher disease burden and service disruptions, rural communities continued to face barriers related to distance, cost, and limited availability of formal healthcare services. Gender and socioeconomic vulnerabilities also influenced healthcare-seeking decisions during this period. Evidence from rural India has also demonstrated healthcare-utilization avoidance during COVID-19 and highlighted the contribution of socioeconomic and demographic factors to healthcare use [6-10].

Although previous studies have documented disruptions in healthcare utilization during the COVID-19 pandemic, less is understood about how individuals perceive changes in their health-seeking behaviour and navigate decisions involving formal healthcare, self-care, and other healthcare options after the pandemic, particularly in urban and rural settings in eastern Gujarat. Quantitative measures can identify patterns of healthcare use but provide limited insights into the reasons, perceptions, and contextual factors underlying these decisions. Furthermore, evidence on these processes among adults in rural and urban settings in eastern Gujarat remains limited. Therefore, this qualitative study explored participants' perceived changes in health-seeking behaviour, healthcare preferences, and decision-making following the COVID-19 pandemic among adults in selected rural and urban settings of eastern Gujarat [11,12].

The study examined post-pandemic health-seeking behaviour among urban and rural populations in eastern Gujarat. It was guided by the following research question: How do adults in selected rural and urban settings of eastern Gujarat perceive changes in their health-seeking behaviour, healthcare preferences, and decision-making following the COVID-19 pandemic? The findings aim to provide insights into perceived changes in health-seeking behaviour following the COVID-19 pandemic.

## Materials and methods

Study design and setting

This qualitative study was conducted as a component of a larger community-based mixed-methods study titled “Assessing the impact of the COVID-19 pandemic on health-seeking behaviour among rural and urban populations of eastern Gujarat.” The qualitative component employed a descriptive design informed by the PASS model of health-seeking behaviour [13].

The study was carried out in eastern Gujarat across both rural and urban settings. The rural setting included selected villages of Sankheda taluka in Chhotaudaipur district, while the urban setting comprised selected localities of Vadodara city.

Study population and period

The study population included adults aged 18-85 years residing in rural areas of Sankheda taluka, Chhotaudaipur district, and urban areas of Vadodara city. The Data collection was conducted over a period of 18 months, from October 2023 to April 2025, occurring after the acute emergency phase of the pandemic. Hereafter, the term “following the COVID-19 pandemic” refers to participants' experiences, perceived differences, and perceptions of healthcare-seeking behaviour during the period after the COVID-19 pandemic. Participants were also asked to reflect retrospectively on their healthcare-seeking practices before the pandemic.

The study was commenced after approval from the Institutional Ethics Committee of Sumandeep Vidyapeeth. Participants were eligible for inclusion if they were 18-85 years of age, had been residing in the study area for at least five years, and provided written informed consent. Individuals who were seriously ill or hospitalized and therefore unable to participate in the interview were excluded from the study. A minimum residence period of five years in the study area was required to ensure that participants had lived in the area before the COVID-19 pandemic and could provide contextual accounts of their health-seeking practices across the pre-pandemic and subsequent periods.

Sampling

Purposive sampling was used to recruit 20 participants, with a pre-planned equal allocation of 10 participants from the urban and 10 from the rural study setting. The equal allocation was pragmatically pre-planned and predetermined, and intended to ensure equal representation of both rural and urban contexts within the qualitative component of the mixed-methods study. Recruitment was not guided by a formal assessment of thematic saturation.

Data collection

Data were collected through in-depth interviews (IDIs) using a semi-structured interview guide, which was developed by the authors for this study (Table 1). Interviews were conducted face-to-face in settings convenient for participants, typically at their homes, to facilitate a comfortable and open environment. Each interview lasted approximately 20-30 minutes and was audio-recorded in Gujarati or Hindi with participant consent.

**Table 1:** Interview domains and questions in the semi-structured in-depth interview guide The semi-structured interview guide was developed by the research team based on the study objectives, relevant literature, and the PASS model, which was used as a sensitizing framework. The guide was pilot-tested and refined before the main interviews COVID-19: coronavirus disease 2019

Interview domain	Interview questions
Domain 1. Health-seeking preferences and decision-making	Can you describe how you usually decide where to seek treatment when you or your family members fall ill?
	Which type of healthcare facility do you usually prefer (government or private)? What are the reasons for your preference?
	Who usually influences or participates in healthcare decisions within your family?
	What factors influence your choice of healthcare providers? Describe in detail the process of decision-making in choosing the healthcare modality
Domain 2. Barriers to seeking healthcare	What were the main reasons you ever delayed or avoided seeking medical care?
	Tell us about a time when you avoided seeking medical care due to anxiety or fear of medical procedures
	Can you share an experience when financial constraints affected your decision to seek medical treatment?
	How do you usually pay for healthcare services and medical expenses, and why?
	What role do government or private health insurance schemes play in your healthcare decisions?
	Did the COVID-19 pandemic create any new barriers to seeking healthcare? If yes, please explain
	Would you be more likely to seek medical care if healthcare costs were lower? Why or why not?
Domain 3. Perceptions of illness and self-treatment	How does the severity of your symptoms influence your decision to seek healthcare?
	Has the COVID-19 pandemic influenced these practices? If yes, how?
	Do you usually try self-treatment (home remedies or over-the-counter medicines) before seeking professional care? Why or why not?
	Have you ever stopped or missed taking prescribed medicines without consulting a doctor? If yes, why?
Domain 4. Medical pluralism	What are your views on traditional healers, Ayurveda, Homeopathy, or other alternative systems of medicine?
	Under what circumstances would you choose these options instead of consulting a doctor?
	What are your views on telemedicine or telephone consultations with doctors?
Domain 5. Changes in health-seeking behaviour after COVID-19	How did the COVID-19 pandemic affect your approach to seeking medical care? (Probe: Have you changed your healthcare facility—for example, from a government hospital to a private clinic, telemedicine, or traditional healers? What influenced this change?
	Has your trust in doctors changed after COVID-19?
	Tell us about any changes in your health habits, such as diet, exercise, or preventive measures, after COVID-19
	How often do you visit a doctor or healthcare provider now compared with before the COVID-19 pandemic?

The interview guide consisted of two sections. The first section captured sociodemographic and socioeconomic characteristics. The second section explored health-seeking behaviours. The semi-structured interview guide was informed by the PASS model of health-seeking behaviour and organized into five broad domains covering health-seeking preferences and decision-making, barriers to healthcare, illness perception and self-care practices, medical pluralism, and post-COVID-19 behavioural changes. The guide was pilot-tested and intended to facilitate open-ended discussions rather than test individual PASS model constructs. The PASS model was used as a sensitizing framework to inform the development of the semi-structured interview guide and its broad domains; it was not used as a deductive coding framework. 

Interviews were conducted by the first author, an MD Community Medicine resident who was self-trained in qualitative interviewing. The interview guide was pilot-tested before the main study and refined accordingly. No repeat interviews or systematic field notes were undertaken, and investigator triangulation was not performed.

Data analysis

All interviews were audio-recorded in Gujarati or Hindi, and transcribed verbatim. Audio recordings were transcribed and translated into English using paid online computer-assisted software. The first author manually reviewed the transcripts line by line and identified meaningful statements relevant to health-seeking behaviour, healthcare preferences, barriers, and decision-making.

Data were analyzed using an inductive thematic analysis approach. Meaningful statements were identified and manually coded, and similar codes were grouped into categories and subsequently organized into themes and subthemes through an iterative process of comparison across transcripts. The final themes were reviewed against the underlying coded data to ensure that they adequately represented participants’ accounts and were interpreted in relation to the study objectives and relevant literature.

Ethical considerations

Ethical approval was obtained from the Institutional Ethics Committee prior to the commencement of the study. Written informed consent was obtained from all participants for participation in the interviews and publication of anonymized findings. Participants were informed about the purpose of the study, the voluntary nature of their participation, their right to withdraw, and the confidentiality of the information provided.

## Results

The qualitative analysis included 20 participants (10 urban and 10 rural) from eastern Gujarat (Table 2).

**Table 2:** Sociodemographic characteristics of qualitative study participants (n = 20) Mean age: 43.6 ± 17.3 years; median: 43.5 years; range: 19-82 years

Characteristic	Category	N	%
Study area	Urban	10	50%
	Rural	10	50%
Sex	Male	17	85%
	Female	3	15%
Age group (years)	18–39	9	45%
	40-59	6	30%
	60-79	4	20%
	≥ 80	1	5%
Occupation	Daily wage labourers	5	25%
	Small-scale business owners	8	40%
	Agricultural workers	4	20%
	Homemakers	3	15%
Educational status	No formal education	14	70%
	Secondary education	5	25%
	Higher education	1	5%
Socioeconomic status (Modified B. G. Prasad classification)	Lower socioeconomic class	15	75%
	Middle/higher socioeconomic class	5	25%
Health insurance coverage	Any insurance (e.g., Ayushman Bharat)	8	40%
	No insurance	12	60%
Family structure	Joint/extended family	20	100%
Household size	4–9 members	20	100%
Residence	Chhota Udaipur district (rural)	10	50%
	Vadodara district (urban)	10	50%

Analysis of the in-depth interviews revealed five major themes related to post-COVID-19 health-seeking behaviour, healthcare preferences, financial barriers, and preventive practices. These themes are presented below with illustrative quotations.

Illustrative quotations are identified using participant codes, where IDI denotes in-depth interview, U represents urban participants, R represents rural participants, and the accompanying number identifies the participant.

Theme 1. Shift in trust and preference toward formal healthcare providers

Participants across both settings described an increased trust in doctors and hospitals following the COVID-19 pandemic. Before the pandemic, many relied on over-the-counter medicines, traditional healers, or delayed care until symptoms worsened. Participants described that heightened fear and awareness prompted earlier consultation with medical professionals.

“We used to take medicines from medical stores… now everyone believes in the doctor.” (IDI-U1). “After Corona… we go to the doctor quickly.” (IDI-U2)

Some urban participants described a noticeable shift toward professional care, whereas some rural participants described continuity with pre-pandemic practices, including established relationships with local providers.

“No change… I used to go to private; I still go to private.” (IDI-R3)

Several participants explicitly stated that the perceived failure of traditional healers during COVID-19 contributed to increased reliance on allopathic care.

“I went to Hakim… got no relief… then I went to the doctor, and it helped.” (IDI-U2)

A perceived preference for private healthcare facilities was observed in both urban and rural settings. Participants cited quicker service, better doctor availability, and perceived quality of care as key reasons for avoiding government facilities.

“Treatment gets late in the government. So, we go to private.” (IDI-U3). “Private medical practitioner is preferred… treatment is quick.” (IDI-R5)

Despite high out-of-pocket expenditure, participants prioritized timely relief, often borrowing money or using savings to access private care.

Theme 2. Affordability, accessibility and healthcare decision-making

Financial hardship emerged as a major determinant of health-seeking behaviour. Participants described delaying care, opting for government facilities, or borrowing money when treatment costs were unaffordable.

“If I don't have the money, then I don't go. I'll go after a while.” (IDI-R2). “I spent about 15,000… eventually had to go to a government dispensary.” (IDI-U6)

Daily wage labourers were particularly vulnerable, often choosing government hospitals despite perceived delays due to cost considerations.

While awareness of government insurance schemes such as Ayushman Bharat was present, utilization remained inconsistent. Insurance was commonly viewed as a resource for major illness rather than routine care.

“Ayushman card is used only if the cost is high.” (IDI-R5)

Participants emphasized that lower healthcare costs would encourage earlier and more complete treatment.

Theme 3. Symptom severity, self-care and medical pluralism

Alternative medicine systems like Ayurveda and homeopathy were generally perceived as slow-acting or unreliable, though a few appreciated their traditional value. Traditional remedies or alternative systems like Ayurveda were often seen as slow-acting or less reliable for serious conditions. One participant said,

"The Ayurvedic treatment, that is only relaxing after a long time... this allopathic gives quick relief" (IDI-U6)

"Medical hospital and its own medicine. We choose them. Medicines work quickly, and Ayurvedic medicines work after a very long time". (IDI-U7)

While some viewed Ayurveda and homeopathy positively, especially for non-urgent conditions. Several participants described greater preference for formal biomedical care. Belief in modern medicine was high, with traditional healers or Ayurvedic/Homeopathic practitioners generally considered slower-acting or not as reliable for immediate relief, although some acknowledged the potential long-term benefits of Ayurveda. Participants mentioned not believing in "Baba with herbs," or that Ayurvedic/Homeopathic treatments were "not instant"

“No belief in Baba or herbs… I take medicine from doctor.” (IDI-R5). “Ayurveda is good, but it works slowly.” (IDI-R2). “Before going to the doctor, I often take home remedies such as granny's remedies or homemade decoctions.” (IDI-R1)

While many preferred formal care, some participants still began treatment with over-the-counter medicine or home remedies, especially when symptoms were mild.

"Occasionally takes medicine from medicine store and also uses home remedies… if do not feel good, then hospital." (IDI-U8). "It seems that the pill does not heal anymore. Then we go to the doctor." (IDI-U1)

However, a section of participants strictly avoided self-treatment:

"No, we go to the hospital first." (IDI-U9). "No, we don’t do home remedies… we go to the doctor." (IDI-U4)

Home remedies like ginger, basil, or turmeric were commonly used in early illness stages, particularly for colds, coughs, and minor fevers.

Symptom severity played a central role in determining care-seeking. Mild illnesses were often managed through home remedies or pharmacy medicines, while worsening symptoms prompted medical consultation.

“If symptoms are mild, we take pills first… if not, then hospital.” (IDI-R5)

Distance to facilities and affordability further influenced the timing and choice of healthcare provider.

Theme 4. Limited adoption of telemedicine

Awareness and use of telemedicine were limited, especially among rural participants. Most preferred face-to-face consultations, citing the need for physical examination, low digital literacy, or lack of familiarity.

“That’s not something I ever tried.” (IDI-R1). “It hasn’t become popular.” (IDI-U5)

A few urban participants viewed telemedicine as useful in emergencies, but not as a substitute for in-person care. Some rural participants described limited familiarity with or access to telemedicine, while some urban participants recognized its potential usefulness in particular circumstances.

Theme 5. Changed preventive and lifestyle behaviors following the COVID-19 pandemic

Regarding general health habits, participants reported increased awareness and adoption of preventive measures following the COVID-19 pandemic, such as washing vegetables, maintaining distance, increasing exercise (like walking), and eating more green vegetables and fruits while reducing outside food consumption. Many participants reported positive behavioral changes following the pandemic. Increased hygiene awareness, dietary improvements, and routine health check-ups were mentioned:

“Now… everyone eats well, everything is covered.” (IDI-U1). “Eating green vegetables brings about a good change.” (IDI-U2). “We eat less food outside… I walk… we have changed our habits since Corona. Discipline life is maintained in eating and diet… we eat less food outside." (IDI-U5)

“Earlier, I did not have any specific timing… now I only eat whatever is prepared at home.” (IDI-R7)

While some reported increased consumption of fruits, vegetables, and reduced tobacco use post-COVID, others saw no significant behavioral change:

“We eat fruits and take seasonal fruits… same as before Corona.” (IDI-R10). “Earlier I used gutkha, now reduced.” (IDI-R5)

However, a few noted improved health awareness, such as hand hygiene or distancing, and reduced use of tobacco or unhealthy foods:

“Used to smoke more bidi… now reduced since Corona.” (IDI-R1). “We now wash hands more, cover mouth when coughing.” (IDI-R2)

Some participants noted increased awareness and adoption of general health habits like washing hands, maintaining distance, and being more cautious if someone coughed. Dietary changes towards consuming more fruits and green vegetables were also reported by some. Interestingly, some participants mentioned a reduction in tobacco consumption since Covid-19. Preventive health practices were inconsistently adopted, influenced by occupation, education, and household priorities.

## Discussion

This qualitative inquiry provides contextual insights into the following COVID-19-era health-seeking behaviour among urban and rural populations in eastern Gujarat (Figure 1). However, the observed urban-rural variations should be interpreted as contextual patterns within the interviewed sample rather than as evidence of systematic differences between urban and rural populations. The findings suggest that the participants described increased health awareness and greater reliance on formal healthcare, while entrenched structural barriers-particularly financial constraints and perceived inefficiencies in public facilities-continue to shape healthcare decision-making.

**Figure 1:**
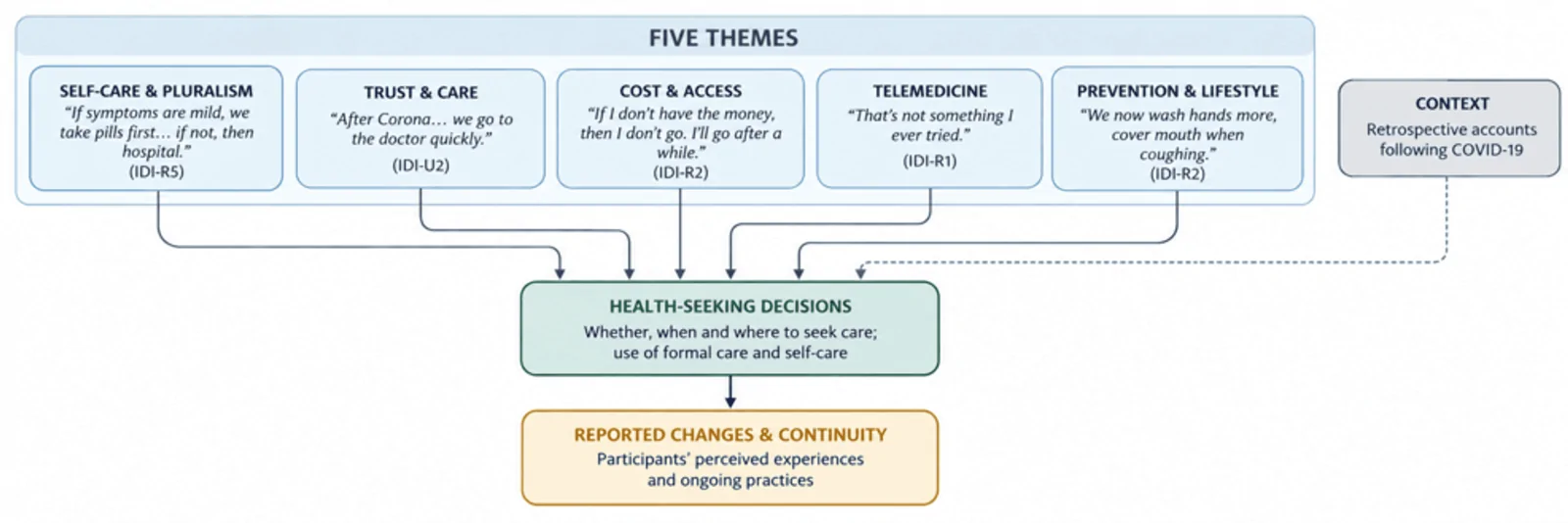
Conceptual framework of perceived health-seeking behaviour following the COVID-19 pandemic COVID-19: coronavirus disease 2019; IDI: in-depth interview (U represents urban participants, R represents rural participants, and the accompanying number identifies the participant)

Heightened risk perception and increased trust in formal care

A central finding was the reported shift toward earlier consultation with doctors, particularly among urban participants. The participants appear to have heightened perceived vulnerability, reduced tolerance for symptoms, and reinforced the perceived superiority of biomedical care over informal or traditional systems. Participants described fear, uncertainty, and the perceived inadequacy of non-formal providers during COVID-19 as key drivers of this transition [14].

This aligns with evidence from other low- and middle-income country settings, where epidemics have been shown to alter risk perception and encourage more timely engagement with formal healthcare systems. However, some rural participants described greater continuity with established healthcare practices, where established relationships with local providers and longstanding care pathways persisted. This suggests that behavioural transformation following public health crises is mediated by contextual realities such as access, familiarity, and affordability rather than by awareness alone [15].

Preference for private healthcare

Despite perceived increased awareness and trust in formal care, participants across both settings expressed a strong preference for private healthcare facilities. Speed of service, doctor availability, and perceived quality were repeatedly cited as reasons for bypassing public facilities. This pattern reflects longstanding systemic challenges within public healthcare delivery, including perceived delays and inconsistent provider presence [16].

Importantly, Participants described a preference for private healthcare even when they recognized the associated financial burden. For some participants, perceived benefits appeared sufficiently important to justify using savings or borrowing money when illness was considered serious. The findings highlight a pragmatic calculus in healthcare decision-making: households weigh cost against perceived effectiveness and speed, often prioritizing immediate relief despite financial strain [17].

Although participants frequently attributed changes in their health-seeking practices to experiences during the COVID-19 pandemic, these changes may not be attributable to the pandemic alone. Broader factors occurring during the study period, including changes in healthcare availability and accessibility, increasing exposure to health information, health-promotion activities, evolving healthcare infrastructure, and greater awareness or use of financial-protection schemes, may also have influenced healthcare preferences and decision-making. Therefore, the findings are best understood as participants' perceptions of changes following the pandemic rather than as evidence of causal effects attributable solely to COVID-19.

Financial constraints in healthcare decision-making

Financial constraints emerged not only as a barrier to healthcare access but also as an important component of healthcare decision-making. Participants described weighing the financial cost of care against perceived quality, waiting time, provider availability, accessibility, and the urgency of their symptoms. 

Participants described delaying treatment, borrowing money, exhausting savings, or switching from private to government facilities due to cost. For daily wage labourers, the economic consequences of illness extended beyond treatment expenses to lost income, further complicating care decisions [18].

These accounts suggest that healthcare choices involved an ongoing trade-off between affordability and the perceived benefits of different care options. The findings therefore indicate that financial protection may influence not only whether care is sought, but also when and from which type of provider it is sought.

Although awareness of government insurance schemes such as Ayushman Bharat was present, their utilization was inconsistent among participants. 

Self-treatment and symptom-based decision-making

The stepwise approach to care-beginning with home remedies or over-the-counter medicines for mild symptoms and escalating to professional care only if symptoms persist-remained common, particularly in rural areas. While such practices reflect culturally embedded traditions and cost-saving strategies, they also raise concerns about potential delays in diagnosis and treatment of more serious conditions [19,20].

Perceived symptom severity functioned as a critical threshold variable in decision-making. This finding reinforces the importance of community-level health education that promotes early recognition of warning signs and appropriate care-seeking pathways [21].

Limited adoption of telemedicine

Although telemedicine gained prominence during the pandemic, its uptake among participants was limited. Digital literacy barriers, preference for physical examination, and limited exposure to virtual platforms constrained adoption, particularly in rural areas. Some participants from the rural setting reported greater limitations in accessing or using telemedicine, while some urban participants recognized its potential usefulness in specific circumstances. However, these findings are based on a small purposive sample and should not be interpreted as representative of telemedicine use across rural or urban Gujarat. These findings suggest that digital health strategies must be accompanied by user education, infrastructure strengthening, and trust-building efforts to achieve equitable integration [18].

Perceived preventive and lifestyle changes following the pandemic

Encouragingly, participants reported increased hygiene practices, dietary modifications, and, in some cases, reduced tobacco use following the pandemic. These behavioural shifts indicate that the participants experienced enhanced health consciousness within households. However, adoption was uneven and influenced by socioeconomic position and daily work demands, raising questions about long-term sustainability.

Medical pluralism

While reliance on traditional healers for serious illness declined, home remedies and alternative systems such as Ayurveda continued to occupy a complementary role for minor ailments and mild symptoms. Participants' accounts reflected medical pluralism rather than exclusive reliance on a single healthcare system. Household remedies and OTC medicines were commonly described as forms of self-care for less severe symptoms, while some participants also reported using traditional healers, Ayurveda, or homeopathy. These practices appeared to coexist with formal healthcare rather than necessarily replacing it, with perceived symptom severity and persistence influencing decisions to seek professional medical care. Following the pandemic, participants appear to have strengthened biomedical trust without fully displacing culturally embedded practices [22].

Implications for policy and health system strengthening

Overall, the findings suggest that following COVID-19 participants experienced increased awareness and trust in professional healthcare but did not eliminate structural inequities. Strengthening public healthcare responsiveness, reducing out-of-pocket expenditure, expanding effective insurance utilization, and investing in rural health infrastructure are critical to sustaining positive behavioural changes. Additionally, risk communication strategies should leverage heightened awareness while reinforcing timely care-seeking for early symptoms [23,24].

By illuminating lived experiences and decision-making trade-offs, this study contributes to a deeper understanding of post-pandemic health-seeking behaviour in a resource-constrained setting and offers insights for strengthening health system resilience in future public health emergencies.

Limitations

This study has several limitations. First, the qualitative sample was relatively small and limited to selected urban and rural areas of eastern Gujarat, which may restrict the transferability of findings to other regions. Second, the sample was predominantly male, with only three female participants, and most participants had no formal education, belonged to lower socioeconomic groups, and lived in joint or extended families. Third, findings rely on self-reported experiences and may be subject to recall bias, particularly when comparing pre- and post-pandemic behaviours. Finally, the interviews were conducted using a pilot-tested semi-structured guide; the researcher's background and interaction with participants may have influenced the data, and no independent parallel coding or investigator triangulation was undertaken. Formal reflexive field notes were not systematically maintained. These characteristics provide important context for interpreting the findings but may limit their transferability to populations with different demographic, educational, socioeconomic, or family characteristics. The experiences reported in this study should therefore not be assumed to represent the health-seeking behaviour of the wider population.

Despite these limitations, the study provides rich, context-specific insights into evolving health-seeking patterns and highlights critical determinants relevant to health policy and service delivery.

## Conclusions

This qualitative study captures the lived experiences of urban and rural participants and contributes valuable evidence on the post-pandemic shift in behavioural health in an Indian setting. Participants described perceived changes in health-seeking behaviour following the COVID-19 pandemic, including greater trust in formal healthcare and increased attention to preventive behaviours. However, financial constraints, perceived delays in public facilities, accessibility barriers, and continued reliance on self-treatment remained important influences on healthcare decisions. The findings provide contextual insights into healthcare decision-making within the selected study settings. Given the retrospective qualitative design, purposive sample, and absence of longitudinal pre-pandemic data, the findings should not be interpreted as evidence of causal effects of COVID-19 or as representative of the wider urban and rural populations. These findings highlight the need for responsive, affordable, and accessible health services and stronger financial protection to support timely healthcare seeking and health-system preparedness for future public health emergencies.

## Appendices

**Table 3:** COREQ 32 item checklist COREQ: Consolidated Criteria for Reporting Qualitative Research

1	Interviewer/facilitator	Authors are listed on p. 1	Interviews were conducted by Dr. Salil Sharma, Senior resident/MD
2	Credentials	Methods, Authors list	M.D community medicine
3	Occupation	Methods, p. 2–3	The interviewer was a Junior Resident
4	Gender	Not reported for the interviewer	Male
5	Experience and training	Methods, p. 2–3	The interviewer had 3 years of experience in qualitative interviewing and relevant self-training
6	Relationship established	Not reported	The interviewer had no prior relationship with participants
7	Participant knowledge of interviewer	Methods, p. 2	Before the interview, participants were informed that the interviewer was a community physician and that the study's purpose was to extract perceived HSB, following COVID-19
8	Interviewer characteristics	The article identifies the author (p. 1), Dr. Salil Sharma, as the interviewer	Dr. Salil Sharma, MD, male, unmarried, Community Physician
9	Methodological orientation and theory	Materials and Methods, p. 2: descriptive qualitative design informed by the PASS model; inductive thematic analysis is described on pp. 2–3	Reported
10	Sampling	Materials and Methods, p. 2: purposive sampling based on predefined inclusion/exclusion criteria; 10 urban and 10 rural participants	Reported
11	Method of approach	Participants were approached face-to-face (Data collection, p. 3)	Reported. “Potential participants were approached face-to-face by the principal investigator at home or in an open environment
12	Sample size	Materials and Methods, p. 2; Results, p. 3: 20 participants, 10 urban and 10 rural	Reported
13	Nonparticipation	Not reported	Not reported
14	Setting of data collection	Materials and Methods, p. 2: face-to-face interviews in convenient settings, typically participants’ homes; study sites described on p. 2	Reported. The exact interview setting for each participant is not specified
15	Presence of nonparticipants	Not reported	Not reported
16	Description of sample	Results, p. 3, Table 2: demographic and socioeconomic characteristics, including area, sex, age, occupation, education, socioeconomic status, insurance, and family structure.	Reported. The table gives broad categories; retain only categories supported by the study records
17	Interview guide	Materials and Methods, p. 2 and Table 1, p. 9: semi-structured guide developed by the authors, informed by PASS; domains and questions are presented.	Reported
18	Repeat interviews	Reported (Data collection, p. 3)	No repeat interviews were conducted
19	Audio/visual recording	Materials and Methods, p. 3: interviews were audio-recorded with participant consent	Reported
20	Field notes	Reported (Data Collection, p. 3)	Field notes were not made
21	Duration	Materials and Methods, p. 3: interviews lasted approximately 20–30 minutes	Reported
22	Data saturation	Reported (Sampling and Data Collection, p. 2)	Sample size of 20 participants was predetermined
23	Transcripts returned	Not reported	Not reported
24	Number of data coders	Materials and Methods, pp. 2–3: coding was manual, and codes/themes were reviewed and refined	Reported
25	Description of the coding tree	Materials and Methods, pp. 2–3: codes were grouped into categories and refined into themes/subthemes; no coding tree or worked example is provided	Reported
26	Derivation of themes	Materials and Methods, p. 2: inductive thematic analysis; Results, pp. 3–8: themes and subthemes are reported	Reported. The PASS model was used as a sensitizing framework to inform the development of the semi- structured interview guide; it was not used as a deductive coding framework
27	Software	Materials and Methods, p. 2: analysis is described as manual; no software was used	Reported
28	Participant checking	Not reported	Not reported
29	Quotations presented	Results, pp. 3–8: illustrative quotations are presented and labeled with participant codes (IDI-U/IDI-R and number); the coding key is on p. 3	Reported
30	Data and findings consistent	Results, pp. 3–8: themes are accompanied by illustrative participant quotations	Reported.
31	Clarity of major themes	Results, pp. 4–6: Five major themes are named and described; theme headings begin on p. 3 and continue through p. 8	Reported
32	Clarity of minor themes/diverse cases	Results, pp. 4–6	Reported
